# TMED family genes and their roles in human diseases

**DOI:** 10.7150/ijms.87272

**Published:** 2023-10-16

**Authors:** Lv Zhou, Huaixu Li, Hui Yao, Xingliang Dai, Peng Gao, Hongwei Cheng

**Affiliations:** Department of Neurosurgery, the First Affiliated Hospital of Anhui Medical University, Hefei 230022, P. R. China.

**Keywords:** TMED, Tumor, Immune response, Diabetes, Neurodegenerative disease.

## Abstract

The members of the transmembrane emp24 domain-containing protein (TMED) family are summarized in human as four subfamilies, α (TMED 4, 9), β (TMED 2), γ (TMED1, 3, 5, 6, 7) and δ (TMED 10), with a total of nine members, which are important regulators of intracellular protein transport and are involved in normal embryonic development, as well as in the pathogenic processes of many human diseases. Here we systematically review the composition, structure and function of TMED family members, and describe the progress of TMED family in human diseases, including malignancies (head and neck tumors, lung cancer, breast cancer, ovarian cancer, endometrial cancer, gastrointestinal tumors, urological tumors, osteosarcomas, etc.), immune responses, diabetes, neurodegenerative diseases, and nonalcoholic fatty liver disease, dilated cardiomyopathy, mucin 1 nephropathy (MKD), and desiccation syndrome (SS). Finally, we discuss and prospect the potential of TMED for disease prognosis prediction and therapeutic targeting, with a view to laying the foundation for therapeutic research based on TMED family causative genes.

## Introduction

Transmembrane emp24 domain-containing protein (TMED), also known as p24 protein, is made up of type I one-way transmembrane proteins that are common in the endoplasmic reticulum (ER), Golgi (Golgi), and ER-Golgi intermediate compartments of the early secretory route and found in all eukaryotes (ERGIC)^1^. Protein transport has been demonstrated to be significantly regulated by members of the TMED family^2^-^5^, and these transport proteins are necessary for both their function in protein-regulated transport and their involvement in structural development throughout embryonic development. In fact, mutations or aberrant protein production in this pathway is a common cause of many human disorders, including cancer and congenital malformations^6^-^9^. This publication provides an overview of existing research on the role of the TMED receptor family in human disease.

## Composition of the TMED family

Encapsulated vesicles and ancillary proteins mediate protein transport in the secretory pathway^10^-^12^. In the early secretory route, coat protein complex II (COPII) mediates protein transport between the endoplasmic reticulum (ER) and the cis-Golgi surface (cis-transport), whereas coat protein complex I (COPI) controls retrograde transport. In membrane-bound vesicles, the COPII-mediated transport chooses well-folded and energetic molecules (cargo) at the ER exit for subsequent export to the Golgi apparatus^15^, ^16^. To handle large volumes of transport cargo successfully, a variety of transport proteins are required, which can total up to 15% of the human proteome^17^. Protein-sorting receptors^12^, transmembrane proteins that convey carbohydrate and/or polypeptide signals in transport cargo and continually cycle between the ER and the Golgi apparatus in vesicular intermediates created by COPI and COPII, are needed to sort a variety of proteins.

**Figure 1:**
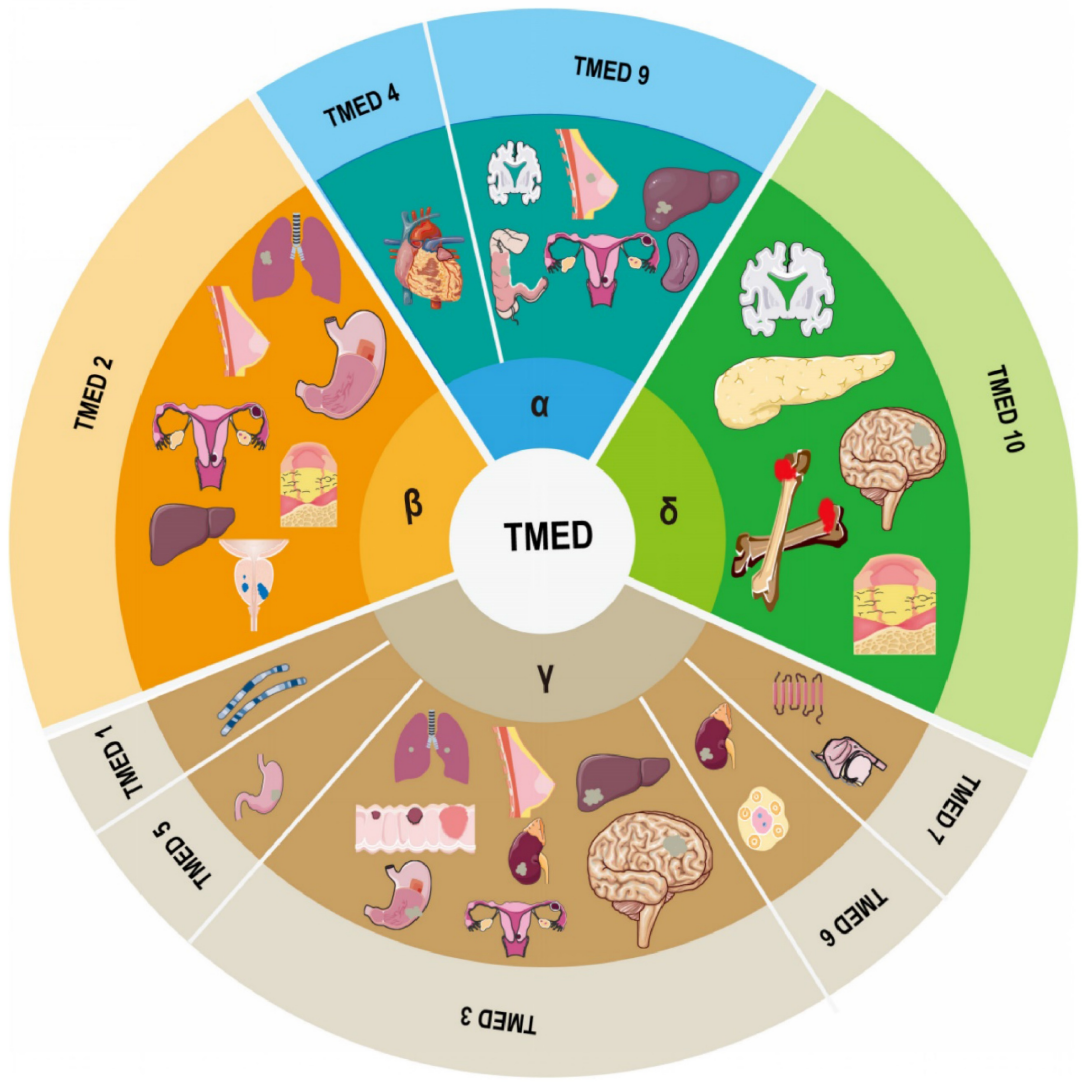
Composition of the TMED family and its involvement in human diseases.

A family of membrane proteins that are abundant in COPI- and COPII-coated vesicles as well as in ER and cis-Golgi membranes shuttles continually between organelles at the center of the early secretory pathway^12^. Members of this family, which are also known as the transmembrane emp24 domain-containing protein (TMED)/p24 family^18^, ^19^, have an average molecular mass of 24 kDa and are consequently frequently referred to as p24 proteins^20^. To date, 10 representatives have been recognized in mammals, of which allelic disruptions in at least two members (TMED2 and 10) have been shown to promote early embryonic death^21^, ^22^. The human genome encodes nine TMEDs; the TMED11 gene incorporates an in-frame stop codon and consequently does not occur in humans, though it has been discovered in other primates^20^. TMED8 is a GOLD domain-containing protein that is not considered a p24 protein due to its size (~300 amino acids) and lack of TM domain and two conserved cysteine residues. It is, however, incorrectly indicated as a TMED/p24 family member in the non-redundant NCBI Protein Data Bank^20^. Thus, the human TMED subfamily includes α (TMED4, 9), β (TMED2), γ (TMED1, 3, 5, 6, 7), and δ (TMED10) members based on phylogenetic analysis (Figure 1).

## Structure and function of the TMED family

All members of the TMED family, while having minimal sequence homology, have the same structural arrangement, which consists of a brief cytoplasmic area at the C-terminus, a flanking region, a coiled-coil domain, a single transmembrane domain, and an N-terminal Golgi-dynamics (GOLD) domain^23^. TMED/p24 members are the only GOLD-containing structural proteins with a globular GOLD domain^19^, ^24^, ^25^.

TMED proteins dimerize and interact with COP protein complexes during cis- and retrograde transport to promote cargo choice and vesicle formation. The GOLD structural domain, assumed to be critical for molecular recognition^26^, has recently been demonstrated to mediate dimerization amongst TMED proteins^23^, ^24^, a function traditionally assigned to the coiled-coil domain. The transmembrane domain has been demonstrated to interact with lipids and contribute to vesicle sprouting^28^, ^29^, contrary to predictions that it would facilitate interactions with intact membrane proteins to control ER channels^27^.

A conserved COP binding motif in the cytoplasmic tail is used to interact with COPI and COPII^30^, ^31^.

Members of the TMED family are involved in the synthesis of COPI vesicles^32^, the creation and organization of early secretory pathway structures (ER and ER-Golgi intermediate compartment)^33^-^35^, and the maintenance of the Golgi apparatus^19^. TMED proteins may potentially serve as cargo receptors for the secretory route, according to certain studies^18^, ^36^. Also, it has been shown that TMEDs carry out a quality control phase by identifying appropriately folded cargo^37^.

Different mammalian p24/TMEDs are involved in different signaling pathways^38^, ^39^, and each molecule acts synergistically to regulate stability. Knockdown or deletion of any of the p24/TMED proteins can result in a loss of expression of other protein members. TMED2 and TMED10 interact with glycoylphatidylinositol (GPI)-anchored proteins to assist in ER entry and translocation of GPIs to the plasma membrane^25^, ^40^, ^41^. TMED2 and TMED10 regulate the stability of one another; when one protein is missing, the amount of the other protein also decreases^5^, ^42^. Another research verified the weak interactions between the GOLD domains of TMED2 and TMED10, which may speed up the assembly and dissociation of the p24 protein complex following the ER^23^. Nuclear magnetic resonance spectroscopy was used to establish how the bottom of the TMED2 GOLD domain interacts with the side of the TMED10 GOLD domain. As a result, Nagae et al suggest that the heterophilic interaction of p24 GOLD domains plays a role in the development of p24 hetero-oligomeric complexes and effective cargo transport^24^. It has been demonstrated that TMED2 deletion causes TMED7 and TMED10 loss as well as decreased TMED9 expression. TMED7 participates in the early secretory pathway by moving molecules from the ER to the Golgi in coordination with TMED2, TMED9, and TMED10^43^.

Members of the p24/TMED protein family are expressed during organ development, with extensive co-expression of the same or different subfamilies of p24/TMED proteins; these proteins are involved in early embryonic development, insulin biosynthesis and subsequent pancreatic β-cell secretion, and amyloid precursor protein metabolism^18^, ^44^. P24/TMED proteins are abundant in the Golgi apparatus of embryonic cells^18^, ^42^, ^45^; different p24/TMED proteins are expressed at different levels. TMED2 and TMED10 are important in mouse embryonic development and aberrant expression of either promotes MC3T3-E1 cell proliferation^5^, ^46^. TMED2 expression was detected in the cytotrophoblast, syncytial trophoblast, and mesenchymal cells of the human placenta at 5.5-40 weeks; TMED2 deficiency resulted in mid-gestational embryonic death, delayed embryonic development, and in absence of the placental labyrinth layer, suggesting TMED2 may be involved in human placental development^47^. In mice, TMED10 deletion reduced TMED9 and TMED3 protein levels, leading to embryonic lethality^21^.

Diseases like cancer can result from abnormalities in the secretory pathway's modulation of protein transport. Particularly, deregulation of the immune system, cancer, liver disease, and pancreatic illness have all been associated with aberrant TMED protein expression. Although the symptoms of various illnesses vary greatly, they all emphasize the need of maintaining stable intracellular levels of TMED proteins. It is difficult to research the family since both too much and too little intracellular TMED might result in similar phenotypes.

## Progress of the TMED family in some common human diseases

### Malignant tumors

Current studies of TMED proteins suggest a function in cell proliferation and differentiation, specifically in malignant tumors. However, our knowledge of how changing TMED levels contribute to the majority of malignancies is still in its infancy. Determining how each TMED protein contributes will be crucial in understanding how many malignancies can form and advance while being promoted or inhibited by TMED proteins.

### Tumors of the head and neck

Head and neck squamous cell carcinoma (HNSC) is the most frequent malignant tumor of the head and neck, occurring largely in the mucosal epithelium of the mouth, pharynx, and larynx^48^. Patients with HNSC are frequently discovered at a late stage and have a poor prognosis because of the tiny dimensions of HNSC lesions and the absence of efficient markers for early diagnosis of tumor growth. The Cancer Genome Atlas (TCGA) and Gene Expression Omnibus (GEO) analysis of the HNSC dataset indicated increased mRNA and protein expression levels of TMED2/9/10 in HNSC. TMED2/9/10 and its co-expressed genes appear to be involved in tumor biological processes such as intracellular transferase complex, protein transport, focal adhesion, and intracellular protein processing, which contribute to malignant behavior, according to analyses from Gene Ontology (GO) and the Kyoto Encyclopedia of Genes and Genomes (KEGG). These findings imply that TMED2/9/10 and related genes may affect HNSC's prognosis as a whole^49^. Genes like X-box binding protein 1 (XBP1) and TMED7 have been identified in transcription factor-gene and protein-protein interaction (PPI) networks as potential HNSC modifiers that may cooperate with TMED2/9/10. Using the HNSC dataset from The Cancer Genome Atlas, Yang et al. discovered differently expressed genes in metastatic and non-metastatic nasopharyngeal cancer cells and found that TMED7 was significantly upregulated in 5-8F cell lines, suggesting TMED7 may be involved in the distant metastasis of nasopharyngeal carcinoma^50^. In addition, Liao et al. found that TMED3 was substantially expressed in gliomas, to be connected to tumor stage, and to have an impact on patient prognosis, according to Liao et al. TMED3 could therefore be a viable immunotherapeutic target and prognostic marker for glioblastoma. 51. Du et al. showed significant interactions between LMAN1 and MCFD2, and F8 and TMED10 by PPI analysis in their study of the expression and clinical characteristics of LMAN1 in gliomas and its effects on cell growth and invasion lead to the hypothesis that TMED10 may interact with LMAN1 to affect glioma cell growth and invasion^52^.

### Lung cancer

Researchers' focus has been drawn to the significance of TMED family genes in the development of lung cancer. TMED proteins have been implicated in the regulation of proliferation and inflammation in lung adenocarcinoma^54^, proliferation, invasion, and therapeutic target sensitivity in non-small cell lung cancer^55^, and invasive and metastatic features in squamous lung cancer^56^.

Feng et al. found that TMED2 knockdown increased apoptosis, inhibited tumor cell proliferation, reduced tumor volume, and decreased tumor biomarker and inflammatory factor levels via TLR4/NF-κB signaling, thereby significantly inhibiting the development of lung adenocarcinoma. Thus, TMED2 may regulate the inflammatory response of lung adenocarcinoma through TLR4/NF-κB signaling and promote the proliferation, development, and prognosis of lung adenocarcinoma by regulating the inflammatory response, providing a new strategy for treatment of lung adenocarcinoma^53^. TMED3 is a protein that is highly expressed in non-small cell lung cancer (NSCLC) tissues, and high TMED3 levels indicate that a patient's chance of survival will be lowered. NSCLC cells that lack TMED3 multiply and invade more slowly and are more vulnerable to the chemotherapy drug cisplatin. TMED3 suppression decreased Wnt/-catenin pathway activity, which was associated with AKT inhibition. The increase of the Wnt/-catenin pathway by TMED3 overexpression was prevented by AKT inhibition. Moreover, the boosting impact of TMED3 overexpression on the proliferation and invasion of NSCLC cells was reversed by the downregulation of Wnt/-catenin activity. *In vitro* tests revealed that TMED3 inhibition has anti-tumor effects in naked mice. Overall, TMED3 stimulates the growth of NSCLC and improves Wnt/-catenin signaling through AKT regulation. The anti-tumor effects of TMED3 inhibition were both strong *in vitro* and *in vivo*, and it may be a promising therapeutic target for NSCLC 54. Xie et al. showed that in lung squamous cell carcinoma (LUSC) tissues, TMED3 expression was elevated, and it was positively linked with tumor development TMED3 knockdown and overexpression both enhanced and inhibited LUSC cell motility, colony formation, and proliferation as well as prevented and promoted apoptosis. In addition, TMED3 knockdown inhibited the epithelial-mesenchymal transition, which is critical for invasion. We further explored downstream targets of TMED3 and identified EZR Gene by RNA sequencing and Ingenuity Pathway Analysis. The formation of LUSC *in vitro* was prevented by EZR knockdown, and TMED3 overexpression's ability to promote LUSC was lessened. Hence, via controlling EZR, TMED3 aids in the formation and progression of lung squamous cell carcinoma 55.

### Breast cancer

Breast cancer is a common malignant tumor in women, with the continuous progress of breast cancer treatment, the survival rate has been greatly improved, but the tumor progression and metastasis are still not optimistic, there have been studies focusing on the role of the TMED family of genes in the process of breast cancer development and provide new biological reference indexes for prognostic assessment of breast cancer and the selection of targeted therapies.

Zhang et al. showed that TMED3 was much more expressed on the mRNA and protein levels in breast cancer tissues and cell lines as compared to healthy controls. TMED3 expression levels were substantially linked with clinicopathological characteristics in breast cancer patients and predicted a poor outcome. TMED3 knockdown prevented the proliferation, migration, invasion, and cell cycle advancement that TMED3 overexpression induced in breast cancer cell lines as compared to controls. TMED3 has also been demonstrated to facilitate breast cancer cell migration and proliferation through Wnt/-catenin signaling^56^. In addition, Pei et al. found that elevated TMED3 levels were significantly associated with ER, PR, Her-2 status, and lymph node metastasis, and were significantly associated with poor overall prognosis. In breast cancer, the microRNA miR-188-3p was discovered to be a new negative regulator of TMED3 57. Ju et al. were the first to report that TMED9 could predict breast cancer prognosis and serve as a therapeutic target. Analysis of collected samples from multiple cohorts and *in vitro* experiments revealed that High TMED9 expression in breast cancer patients encouraged the growth and migration of breast cancer cells and predicted a poor prognosis^58^. When compared to samples from healthy breasts, TMED2 was markedly elevated in breast cancer patients. Moreover, increased TMED2 mRNA expression was substantially linked to worse overall survival (OS) in all breast cancers as well as in patients with the luminal A, luminal B, or ER-positive subtypes of the disease. The relationship between higher TMED2 protein levels and lower OS was verified by Western blotting. Patients with breast cancer who expressed more TMED2 had considerably worse prognoses. As TMED2 is oncogenic, we propose that it may be a promising therapeutic target for breast cancer^59^.

### Malignant tumors of the female reproductive system

Differential expression of TMED family genes in relation to the outcome for individuals with endometrial, and cervical cancers has attracted equal attention from researchers. Findings on the progression and prognostic function of different TMED family members in different female genital malignancies has provided a basis for exploring novel therapeutic targets.

One study discovered that ovarian cancer patients had higher TMED2 levels, and that ovarian cancer cells that overexpressed TMED2 had greater cell migration and proliferation. These findings imply that TMED2 may have a role in the development of ovarian cancer^60^. A recent study confirmed that high TMED9 expression was correlated to progression, plasma cell type, and inferior histological grade in epithelial ovarian cancer, which demonstrates potential predictive value for both disease-free and overall survival. More in-depth functional studies revealed that TMED9 knockdown decreased epithelial ovarian carcinoma (EOC) cells' migration, invasion, cell proliferation, and colony formation, indicating TMED9 might be a useful prognostic biomarker and supporting the idea that targeting TMED9 might be an innovative approach to treating EOC^61^.

The most common gynecological cancer in developed nations, endometrial cancer (EC), is growing every year^62^. According to Zhang et al., downregulation of TMED3 resulted in an inhibition of the EC cell cycle, growth, and migration but a promotion of apoptosis. Additional *in vivo* tests supported the finding that TMED3 knockdown inhibits tumor growth. Through the upregulation of pro-apoptotic proteins and the targeting of the PI3K/AKT signaling pathway, TMED3 deletion may decrease cell viability, according to an investigation of the molecular pathways. The malignant phenotype of EC cells may be impacted by TMED3 knockdown, which could slow tumor growth and provide information for the creation of targeted drugs for the treatment of EC^63^.

Latest evidence has shown that TMED5 promotes tumors by activating Wnt7b/-catenin signaling during the aggressive development of cervical cancer^64^. Circ 0018289 has been found to indirectly control cervical angiogenesis and carcinogenesis through miR-183-5p while miR-183-5p directly targets TMED5 and inhibits it. TMED5 knockdown exhibited the same angiogenesis and cervical cancer suppression phenotypes as miR-183-5p overexpression. Furthermore, the CIRC_0018289/miR-183-5p/TMED5 axis promoted elevated expression of two angiogenic inducers, VEGFA and FGF2, leading to tumor-associated angiogenesis. It has been established that the CIRC_0018289/miR-183-5p/TMED5 regulatory network represents a novel molecular basis for controlling cervical carcinogenesis^65^. In addition, TMED2 may be crucial in the initiation and advancement of squamous cervical carcinoma because of higher TMED2 expression in this type of cancer and its substantial association with worse overall and recurrence-free survival, according to a multi-omics investigation^66^.

### Tumors of the digestive system

In a multi-omics bioinformatics analysis, TMED2 was found to be a common diagnostic and prognostic biomarker in patients with different clinicopathological features of esophageal adenocarcinoma. It has been hypothesized that TMED2 promoter hypermethylation is closely associated with its overexpression in esophageal adenocarcinoma^66^.

The third most common cancer that leads to death worldwide is gastric carcinoma. It remains unclear why obese individuals have a higher chance of acquiring gastric carcinoma. In obesity, serum leptin levels are high and *in vitro* studies have shown that serum leptin can induce proliferation of gastric carcinoma cells. However, to date, no *in vivo* tumorigenic effects of leptin on the stomach have been reported. Thus, the *in vivo* leptin-induced gastric carcinoma experiment designed by Isyraqiah et al. in rats, which showed upregulation of numerous potential driver genes such as TMED2, is of interest^67^. Peng et al. found that miR-876-3p expression was downregulated in gastric carcinoma, and its mRNA level was negatively correlated with cisplatin resistance in gastric carcinoma. Also, the decreased expression of miR-876-3p revealed that patients with gastric cancer had a bad prognosis. TMED3 was identified by luciferase and biotin-miRNA pull-down tests as a direct target of miR-876-3p. TMED3-targeted small interfering RNA (siRNA) reduced the impact of miR-876-3p on the stem cell-like properties of SGC-7901/DDP cells and their resistance to cisplatin. By targeting TMED3, miR-876-3p increases cisplatin sensitivity and reduces stem cell-like characteristics in gastric cancer. TMED3 may therefore be a potential therapeutic target for gastric cancer^68^. TMED5 has also been shown to be upregulated in gastric carcinoma tissues. Direct targets of miR-27b-3p are TMED5 and TP73-AS1, and miR-27b-3p has a negative correlation with both of these genes. By sponging miR-27b-3p to control TMED5, P73-AS1 has been found to encourage GC proliferation, migration, and invasion^69^.

Previously, it was discovered that TMED3 inhibits metastasis in human colorectal cancer (CRC) cells via way of the WNT-TCF pathway^70^. TMED9 was more than two-fold increased when TMED3 was knocked down in colon cancer cells. It is believed that TMED3-mediated WNT signaling prevents metastasis by blocking TMED9. In a separate study, TMED3 deletion increased TMED9 levels, extended TGF-signaling, and upregulated genes with migratory and invasive effects; these effects were comparable to those shown when WNT signaling was inhibited^71^. During cancer development, TMED proteins form regulatory loops with one another to regulate their levels. Thus, TMED9 functions as an oncogene in colon cancer, and the malignant properties of TMED3 are cell-type specific. However, Wang et al. showed that elevated TMED3 expression in CRC correlated with patient survival outcome, suggesting TMED3 may be a prognostic biomarker for this type of cancer^72^. The importance of TMED3 in CRC therefore remains largely elusive.

TMED2 is the only member of the mammalian β subfamily that shows cell type selectivity in cancer^46^, ^60^. In hepatocytes, TMED2 is necessary for normal cell proliferation, as evidenced by the fact that 99J mutant pure mice's embryos were smaller than those of their littermates before they died in the middle of pregnancy and therefore that TMED2 is necessary for normal cell proliferation^22^. In addition, heterozygotes were more likely to develop hepatocellular carcinoma (HCC) than wild-type siblings^4^. Thus, in hepatocytes, a decrease in TMED2 led to an increase in liver tumorigenesis, demonstrating that TMED2 may have tumor suppressor-like properties. Contrarily, low expression of TMED3 prevented hepatocellular carcinoma cells from migrating, whereas overexpression of TMED3 increased cell viability. Because TMED3-overexpressing cells had higher amounts of IL-11 and STAT3 phosphorylation, it is believed that TMED3 encourages metastasis in hepatocytes^73^.

TMED9 levels in HCC tissues were found to be enhanced by proteomic analysis. In HCC tissues as opposed to healthy liver tissues or precancerous lesions, TMED9 mRNA and protein levels were greater. In individuals with HCC, TMED9 mRNA expression levels were substantially correlated with advanced stage and poor prognosis. Moreover, vascular invasion and disease-free survival were favorably linked with TMED9 protein expression levels. Manipulation of TMED9 expression in HCC cells *in vitro* significantly affected cell migration, invasion, proliferation, and colony-forming ability^74^.

### Tumors of the urinary system

Prostate cancer is a common malignancy in men. By analyzing 498 cases of differentially expressed prostate cancer genes in TCGA, Chen et al. found that TMED2 and TMED10 were downregulated in prostate cancer tissues and were biomarkers of good prognosis, as their high expression was linked to good OS in prostate cancer patients, revealing the potential role of TMED2 and TMED10 in prostate development, prognosis, and possible treatment^75^. Another study using a combination of high-throughput transcriptomic and RNAi technologies identified TMED3 as a possible therapeutic target for prostate cancer^76^.

TMED3 differential expression was found between clear cell renal cell carcinoma (RCC)patients with low stage (I and II) and high stage (III and IV) in the TCGA and ICGC cohorts, as well as between RCC patients with low stage (I and II) and high stage (III and IV) in the TCGA cohort. TMED3 overexpression has been linked to a poor prognosis in RCC patients. TMED3 was further substantiated as a potential prognostic marker for RCC by Kaplan-Meier survival analysis, multivariate analysis, and time-dependent area under the curve (AUC) of the Uno c index^77^. A particularly aggressive cancer that mostly affects adolescents and young adults is TFE3-translocated RCC. The TFE3 transcription factor on chromosome Xp11.2 had a translocation event, which led to the pathogen-specific molecular abnormalities of this subtype^78^. Pflueger et al.^79^ showed that TMED6-COG8 may be a new molecular tumor marker for TFE3-translocated RCC because it was found to be significantly higher in TFE3-translocated RCC when compared to clear cell and papillary RCC.

### Other malignancies

Osteosarcoma is a primary cancer that primarily affects children and young people. Xu et al. found that TMED3 expression was significantly higher in osteosarcoma tissues than in matched adjacent normal tissues. *In vitro* studies revealed that TMED3 knockdown prevented the growth of osteosarcoma by reducing proliferation and migration and increasing apoptosis, whereas TMED3 knockdown prevented the formation of osteosarcoma *in vivo*. Furthermore, protein S15A (RPS15A) was discovered to be a downstream target of TMED3 that contributes to the development of osteosarcoma. Further research revealed that the inhibitory effect on osteosarcoma cells was enhanced by simultaneous knockdown of RPS15A and TMED3. Notably, the pro-moter impact of TMED3 overexpression in osteosarcoma cells was reduced by downregulating RPS15A. These results demonstrate the critical role of the TMED3/RPS15A axis in tumor growth and suggest a potential molecular target for osteosarcoma treatment^80^. TMED3 has also been proposed as a potential therapeutic target for the treatment of chordoma, a rare low-grade bone axis tumor; downregulation of TMED3 expression decreased chordoma cell survival and migration and increased apoptosis^81^. Ge et al. discovered that MM.1S and RPMI8226, two multiple myeloma cell lines, had high levels of TMED2 expression, and that silencing TMED2 resulted in a significant drop in viability and an increase in apoptosis, indicating TMED2 may be crucial in the pathogenesis of multiple myeloma 82.

As the cargo and structural proteins between endoplasmic reticulum and Golgi apparatus, the abnormalities of TMED family genes will directly affect the process of protein transport and related pathways, which will lead to the development of malignant tumors. According to the previous literature, we have found that TMED family proteins participate in several classic tumor pathways, such as Wnt/-catenin pathway, PI3K/AKT signaling pathway, WNT-TCF pathway, and so on. The study of TMED family genes can provide us with new perspectives on the mechanism of tumor research, and lay the foundation for clinical translation and even targeted therapy research.

## TMED genes in immune response

Although it is well known that proteins with GOLD domains are involved in vesicular transport, a number of recent articles have proposed new functions for these proteins. Emerging data on the potential function of TMED family members in innate immune modulation is particularly intriguing.

Via influencing the translocation of ST2 receptors (members of the Toll-like/IL-1 receptor superfamily) to the cell membrane, *in vivo* investigations have demonstrated that TMED1 is involved in IL-33 signaling^47^. By boosting neutrophil counts at the site of infection and speeding up bacterial clearance, IL-33 alleviates sepsis^83^. In addition to interacting with IL-33 to produce T-cell mediated immune responses, ST2 receptors also have a role in the development of infectious illnesses, asthma, and allergy reactions^84^, ^85^. The ST2 receptor's TIR structural domain and TMED1's GOLD domain interact with one another in the IL-33 signaling pathway^47^. TMED members are, at least indirectly, involved in immune system signaling pathways and cell differentiation because TMED1 is interestingly also implicated in the expression of other ILs, such as IL-8 and IL-6^48^.

TMED2 is required for the cellular interferon (IFN) response to viral DNA varies depending on MITA (facilitator of IRF3 activation, also known as STING). plays a critical role in the innate immune response to cell-soluble viral double-stranded DNA. Assembly of COPII facilitates MITA translocation; TMED2 regulates MITA signaling and enhances the anti-DNA virus immune response in a number of ways, including enhanced MITA dimerization, translocation to the ER, and transport from the ER to the perinuclear region. Inhibition or deletion of TMED2 in cells significantly increases herpes simplex virus 1 (HSV-1) titer volume and results in impaired IFN-1 production upon HSV-1 infection^86^.

TMED7, a member of the γ subfamily, is important in the negative control of Toll-like receptor (TLR) 4 signaling, a receptor for the bacterial product endotoxin, which is regulated by sending signals, biogenesis, and transportation. TMED7 is necessary for TLR4 to form a stable complex with its outer structural domain during its translocation from the ER to the cell surface via the Golgi. TLR4 is involved in activating many downstream intracellular pathways, including the NF-κB pathway in response to cellular stress^87^. Severe inflammatory disorders like sepsis can develop as a result of abnormal TLR4 signaling^36^. Another study shown that miR-340-5p overexpression reduced the harm that Mycobacterium TB infection caused to A549 cells by activating the TMED7/NF-B axis^88^. After lipopolysaccharide (LPS) stimulation of human monocytes, TMED7 exhibits a biphasic rise. TMED7 was also primarily localized to nucleosomes which are the basic structural units of chromatin formed by DNA and histones and the Golgi apparatus, with enhanced localization to late nucleosomes after LPS stimulation. The TRIF/TRAM complex cannot form properly due to homotypic interactions between TMED7 and TAG, which also block MYD88-independent TLR4 signaling. TMED7 may therefore be a crucial suppressor of innate immunological signaling and TLR4^48^.

In summary, these studies suggest that further analysis of the role of the TMED family in regulating the IL1R/TLR family may provide new insights into the control of innate immunity. The therapeutic implications of these data may lie in the control of clinical inflammatory immunity and improvement of vaccine adjuvants.

### Diabetes

TMED6 participates in protein transport and secretion and is specifically expressed in pancreatic islets. Thus, TMED6 has a potential role in the control of insulin vesicles^89^. In the pancreas, TMED6 mRNA is strongly and specifically expressed. TMED6 expression was only found in pancreatic islets, according to immunofluorescence of the mouse pancreas; expression was higher in cells than cells. TMED6 gene expression can be knocked down in cells to decrease insulin production at Min6. In addition, diabetic Goto-Kakizaki rats had considerably less TMED6 gene expression. TMED6 may therefore play a crucial role in islet biology, particularly when it comes to the production or secretion of hormones, and its dysregulation may be linked to the onset of diabetes^39^. Further supporting this view, a global genome and transcriptome analysis of human islets revealed new genes affecting glucose metabolism, including TMED628. TMED10 is located on the pancreatic -cells' plasma membrane and is necessary for the folding, grading, and release of insulin^90^-^92^. It has been demonstrated that Sdf2l1 controls ERAD through interactions with the transport protein TMED10. In response to ongoing ER stress, inhibition of Sdf2l1 expression in the liver causes insulin resistance and raises triglyceride levels. Restoration of Sdf2l1 expression in obese and diabetic mice improved glucose tolerance, reduced fatty liver, and lessened ER stress. In diabetic individuals, insufficient Sdf2l1 induction was linked to insulin resistance and steatohepatitis development. Hence, the inability to develop TMED10-mediated ER stress responses in the liver may contribute to non-alcoholic steatohepatitis and obesity-associated diabetes^93^.

Finally, five new single nucleotide polymorphisms (SNPs) were identified using clinical and genetic risk factors to predict the incidence of type 2 diabetes in Koreans. One of the corresponding genes, SNP rs11057302, is located on an intron of TMED2, suggesting TMED2 may be involved in the involvement of type 2 diabetes^94^, although further experimental validation is needed.

In conclusion, the above studies suggest that TMED family members TMED6 and TMED10 may play an important role in the development of diabetes mellitus, for the TMED family in the mechanism of diabetes mellitus in the future may bring new opportunities for the treatment of diabetes mellitus, in addition to the TMED2 may be a potential target for the study of diabetes mellitus, which deserves our attention.

### Neurodegenerative disease

Amyloid β (Aβ) peptides accumulate extracellularly in the brains of people with Alzheimer's disease. The cleavage of the amyloid precursor protein by -secretase results in the production of Aβ, which impairs neurological function. TMED10 was previously known as a γ-secretase regulatory protein, and although TMED10 is expressed throughout the brain, it is most highly expressed in the hippocampus, an area of the brain essential for learning and memory and where AD pathology is typically observed. Interestingly, TMED10 levels are highest during embryonic development and decline with age. In humans, total TMED10 levels are lower in the frontal cortex and hippocampus of patients with Alzheimer's disease^95^, where downregulated TMED10 induces autophagy through activation of autophagy-associated gene 4B cysteine peptidase (ATG4B)^96^. TMED10 has been demonstrated in mice studies to be broadly expressed in the brain's grey matter, however it was primarily localized at the presynaptic membrane where neurons are connected. The presence of TMED10 at presynaptic junctions supports a role in Aβ secretion because amyloid precursor processing enzymes, such gamma-secretase, are similarly localized to the presynaptic membrane^97^, ^98^. TMED10 also prevents the secretory route from carrying amyloid precursor protein, which reduces the buildup of fully processed amyloid precursor protein. TMED9 also lessens Aβ buildup in the brain^99^. TMED9 and TMED10 may thus be crucial in halting the progression of Alzheimer's disease.

Recent research has linked abnormal post-translational modifications (PTMs) to the pathophysiology of schizophrenia. A sophisticated class of glycolipids known as GPI binds glycoproteins and surface proteins to the cell membrane. One of the most frequent PTMs is the binding of GPI to proteins, and GPI-associated proteins (GPI-APs) support a variety of cell surface activities, including the formation and maintenance of synapses. Mutations in GPI processing pathways are linked to intellectual disability, suggesting a possible involvement for GPI-APs in cognition and cognitive dysfunction in schizophrenia. TMED10 specifically recognizes and is essential for quality control of GPI-APs. Reduced TMED10 protein expression in schizophrenic patients suggests abnormal regulation of GPI-AP output by the ER^100^. TMED10 is also involved in the regulation of unconventional secretion and aggregation of mutant Huntington's proteins by GRASP55, providing important insight into the progression of Huntington's disease^101^.

Taken together, the available literature suggests that only TMED10 and TMED9 of the TMED family seem to be closely associated with neurodegenerative diseases, which is worthy of consideration as to whether it suggests that our research in the TMED family of genes in neurodegenerative diseases should be focused on these two genes.

### Other systemic diseases

TMED2 is required for normal hepatocyte proliferation^22^. TMED2 is necessary for the health of the liver, according to histological and molecular analyses of the livers of mice with the 99J mutation, or Tmed299J/+ heterozygotes. Expanded ER membranes and higher eIF2 levels were seen in the isolated livers of heterozygous mice, indicating that ER stress and the PERK unfolded protein response had been activated. At 6 months of age, a histological examination of mouse livers revealed that 28% of heterozygous mice had non-alcoholic steatohepatopathy-like clinical signs.

TMED2 may therefore contribute to the emergence of non-alcoholic fatty liver disease^4^.

A Pakistani study identified a deleterious variant of TMED4 that resulted in gene overexpression in myocardial tissue of patients with dilated cardiomyopathy, suggesting a possible involvement of TMED4 in myocardial dilatation^102^.

A shift mutation (MUC1-fs) in the MUC1 gene, which is therefore trapped in vesicles harboring cargo receptors in the TMED9-mediated early secretory pathway, causes Mucin 1 nephropathy (MKD), a neutrophil protein disease. When the tiny molecule BRD4780 attaches to TMED9, it releases MUC1-fs and sends it on its way to be degraded by lysosomes. By promoting lysosomal degradation and targeting TMED9, this small molecule reverses proteinopathies and demonstrates the potential therapeutic value of TMED9 in proteinopathies 103.

Disturbances in multiple signaling pathways involved in salivary secretion can cause xerostomia linked with Sjögren syndrome (SS), which is a complex condition. Patients with SS xerostomia have considerably lower levels of TMED10, PDIA4, and CanX gene expression compared to healthy controls, but not statistically different from SS non-xerostomia patients, demonstrating that TMED10 gene expression is reduced in xerostomia patients by a different mechanism than in Alzheimer's disease patients and that SS-associated xerostomia is not caused by increased autophagy activation^104^. TMED10 might be significant in the development of osteoarthritis^105^, but more research is required.

## Summary and outlook

Members of the TMED family are type I transmembrane proteins that play important roles in Golgi-ER bidirectional transport. Recent studies of its members have shown that variations in TMED genes can lead to developmental abnormalities and disease progression in humans. Although targeted therapeutic agents for the TMED family have not yet been developed, studies suggest that members of the TMED family may provide a reference for prognostic prediction and therapeutic targets, laying the foundation for further translational medicine research.
